# Effects of physical and chemical pretreatments on drying and quality properties of blackberry (*Rubus* spp.) in hot air dryer

**DOI:** 10.1002/fsn3.1678

**Published:** 2020-06-08

**Authors:** Mohammad Kaveh, Ebrahim Taghinezhad, Muhammad Aziz

**Affiliations:** ^1^ Department of Biosystems Engineering College of Agriculture and Natural Resources University of Mohaghegh Ardabili Ardabil Iran; ^2^ Department of Agricultural Engineering and Technology Moghan College of Agriculture and Natural Resources University of Mohaghegh Ardabili Ardabil Iran; ^3^ Institute of Industrial Science The University of Tokyo Tokyo Japan

**Keywords:** blackberry (*Rubus* spp.), pretreatment, quality, specific energy consumption

## Abstract

This research examines the impact of various pretreatments on effective moisture diffusivity coefficient (*D_eff_*), activation energy (*E_a_*), specific energy consumption (*SEC*), color, and shrinkage of blackberry (*Rubus* spp.). Hot air drying experiments were conducted under three different temperatures (50, 60, and 70°C) and four pretreatments, including thermal pretreatment by hot water blanching at 70, 80, and 90°C, pulse pretreatment with microwave having power of 90, 180, and 360 W, chemical pretreatment using ascorbic acid (1% in distilled water), and mechanical pretreatment using ultrasonic vibration with working frequency of 28 ± 5% kHz for 15, 30, and 45 min. The results show that the highest *D_eff_* value, which was 1.00 × 10^–8^ m^2^/s, could be achieved by using a microwave pretreatment with power and drying temperature of 360 W and 70°C͘, respectively. Moreover, the lowest *D_eff_* value obtained from this similar pretreatment condition was 3.10 × 10^–9^ m^2^/s at a drying temperature of 50°C, while *E_a_* ranged from 13.61 to 26.02 kJ/mol. The highest and lowest *SECs* were 269.91 kW hr/kg for the control sample and 75.63 kW hr/kg for the microwave pretreatment, respectively. Furthermore, the largest color change and shrinkage were detected in ascorbic acid pretreatment and control sample, respectively.

## Graphical abstract

Hot air drying experiments have been conducted under three different temperatures and four pretreatments, including pretreatments using thermal blanching, pulse, chemical, and ultrasonic vibration. Then, drying kinetics, quality, and energy consumption of blackberry in hot air dryer were studied.

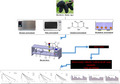

## 1 INTRODUCTION

Blackberry (*Rubus* spp.) is a small spherical fruit grown in Asia (including Iran), Europe, North America, and the temperate regions throughout the world. Blackberry is a rich source of natural antioxidants, including anthocyanins and phenolic acids (López‐Vidaña et al., 2019; Nogueira, Fakhouri, & de Oliveira, 2019). Blackberry fruit has a lot of health benefits for human beings, such as protection against liver damage and lowering blood pressure (Cervantes, Lincon, Soto, Roldan, & González, 2016), strong anti‐inflammatory, antimicrobial activities (Ferrari, Germer, & de Aguirre, 2012), and also an annihilator of human cancer cells (Yamashita et al., 2017).

Drying or dehydration of agricultural products reduces both chemical reactions and microorganism activities, and increases the preservation time of these products (Defraeye, 2014). Drying of agricultural products is conducted by removing the moisture through different methods, including heating (thermal, infrared, microwave, vacuum, solar, etc.) and water extraction from the products (Sun, Zhang, & Mujumdar, 2019). In general, drying process has two stages. At the first stage, the product still has high moisture content which is distributed thoroughly in inner side and surface. At this stage, moisture is available as a free surface water, and hence, drying in this stage can be considered as surface evaporation. As drying is proceeding, the second stage of drying begins and moisture is transferred from the inside to the outside of the product surface where the moisture is evaporated (Schössler, Jäger, & Knorr, 2012). The required energy in the second stage is significantly larger compared to the energy required during the first stage of drying (surface evaporation) (Dibagar & Amiri Chayjan, 2019). The various dryers have been used for blackberry drying, such as vibro‐fluidized bed, vacuum (Giraldo Gomez et al., 2011), spray (Ferrari et al., 2012), solar (López‐Vidaña et al., 2019), freeze (Yamashita et al., 2017), and convective drying with ultrasound pretreatment (Romero & Yépez, 2015). However, to the best knowledge of the authors, there is no report investigating the physical and chemical pretreatments for blackberry by hot air dryer.

It is important to note that the changes during drying of product with high sugar content (like blackberry) lead to significant changes in texture, color, and taste. Therefore, it is necessary to minimize these changes to the lowest extent, including the adoption of appropriate pretreatments (Ramachandran, Akbarzadeh, Paliwal, & Cenkowski, 2018; Wang et al., 2018). Applying different pretreatment stage before the main drying process can effectively increase the mass convection speed (dehydration) and reduce the drying time, as well as specific energy consumption (*SEC*). Pretreatments are expected able to form fast expansion and contraction alternatively in the samples, similar to what a sponge does. Forces that are involved in this mechanical mechanism create microscopic ducts which can lead to displacement and moisture transfer to the outer layers of products (Chemat et al., 2017; Rodríguez et al., 2019). Many researches related to the effect of various pretreatments on drying time, effective moisture diffusivity coefficient (*D_eff_*), activation energy (*E_a_*), *SEC*, color, and shrinkage, for the drying of various agricultural products and foods have been done. These include almond (Kaveh, Jahanbakhshi, Abbaspour‐Gilandeh, Taghinezhad, & Moghimi, 2018), potato (Rojas & Augusto, 2018), parsley leaves (Sledz, Wiktor, Rybak, Nowacka, & Witrowa‐Rajchert, 2016), chayote (Akonor & Tortoe, 2014), wed (Adedeji, Gachovska, Ngadi, & Raghavan, 2008), and apple (Motevali & Zabihnia, 2017).

Different research studies have tried to calculate $D_{\text{eff}}$ , $E_{a}$ , SEC , color, and shrinkage of different types of products with different pretreatments. There is no comprehensive research which has been carried out so far related to different levels of various pretreatments on blackberry. Hence, the objective of this research is checking the effects of various pretreatments, including thermal (blanching), pulse (microwave), mechanical (ultrasonic), and chemical (ascorbic acid), on the drying time, $D_{\text{eff}}$ , $E_{a}$ , SEC , color, and shrinkage during drying of blackberry in hot air dryer.

## 2 MATERIAL AND METHODS

### 2.1 Blackberry preparation

The blackberry fruit was obtained from Mahabad City bazar in West Azerbaijan province, Iran. After being harvested and separated, blackberries were stored in refrigerator at a temperature of 3°C. However, before the experiments, blackberries were kept in the room temperature for 1 hr. Moisture content of the samples was measured by employing an oven‐drying method (Memmert INB200, Memmert GmbH + Co. KG, Schwabach, Germany) for 24 hr and at temperature of 70°C (López‐Vidaña et al., 2019). The initial moisture content of samples was 4.23 ± 0.5 wt% on dry basis (db).

### 2.2 Pretreatments

#### 2.2.1 Blanching pretreatment

To remove the enzymatic browning reactions, blanching operation was applied to all the samples before applying the pretreatments. Various durations and temperatures were suggested for this operation. After comparing with the previous results, 3 min at 70°C, 2.5 min at 80°C, and 2 min at 90°C were selected as each combination of duration and temperature for blanching, respectively.

#### 2.2.2 Microwave pretreatment

For the microwave pretreatment, a microwave device (Sharp R‐861SLM) with a frequency of 50 Hz and maximum thermal power of 900 W, which is configurable in its power (90, 180, 360, 600, and 900 W), was used. The samples were put in the microwave oven, and the microwave pretreatment was carried out at 3 levels of power, which are 90, 180, and 360 W, for 10, 5, and 2.5 min, respectively. It should be noted that the conditions for applying a microwave pretreatment are based on the same SEC value. Therefore, by applying these power levels for each corresponding treatment durations, the amount of SEC due to the microwave was in the same value for all treatments (Motevali & Zabihnia, 2017).

#### 2.2.3 Ultrasonic pretreatment

To pretreat the samples with ultrasonic waves, an ultrasonic bath machine (PARSONIC 7500S, Pars Nahand Company) was used with inner dimensions of 24 × 13.5 × 10 cm^3^ (volume of 2.6 L). This devise is capable of producing ultrasonic waves with frequency and power of 28 kHz and 70 W, respectively. The bath tank of ultrasonic was filled with 2 L of distilled water, and the samples were exposed to ultrasonic waves for three different durations (15, 30, and 45 min) in 30°C temperature. The experiments for each condition were replicated for three times.

#### 2.2.4 Ascorbic acid

At the beginning, ascorbic acid was diluted in a distilled water with dilution ratio of 1%. Furthermore, the samples were immersed in the solution for 2 min at a temperature of 22°C (Ali Motevali & Hashemi, 2018; Doymaz, 2004).

### 2.3 Hot air drying

**FIGURE 1:**
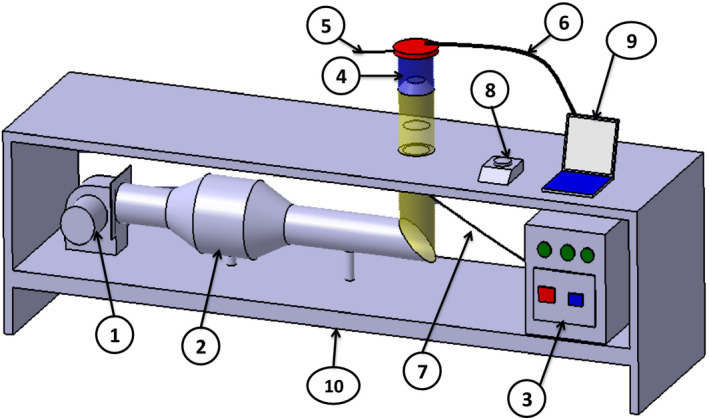
Schematic picture of laboratory scale convective hot air drying: (1) fan and electro motor, (2) electrical heater, (3) control panel, (4) drying chamber, (5) air velocity recorder, (6) outlet air temperature recorder, (7) input air temperature recorder, (8) digital balance, (9) computer, (10) chassis

The 200 g samples were placed in the middle of the duct of hot air dryer. The samples were weighed by using a digital scale (GF‐6000, A&D Co. Ltd.) with an accuracy of 0.01 g before starting the drying experiment (Figure 1). The laboratory dryer had a centrifugal blower which transfers and blows the hot air in a parallel direction to the material's substrate. Before the experiment, the device was initially conditioned for 20 min until the temperature and the air velocity of the dryer become stable. Then, the samples were arranged on the dryer's substrate. Temperature and relative humidity inside the dryer are determining variables in drying of foods, and therefore, in each experiment, they were measured and recorded by using a combined digital thermometer and hygrometer (Lutron TM‐903, Taiwan) with accuracies of ±0.1°C and ±3% RH, respectively. During the drying experiment, the ambient temperature and relative humidity had the average values of 20 ± 4°C and 15 ± 5%, respectively. Experiments were performed at three asir temperature levels (50, 60, and 70°C) and an airflow speed of 1 m/s. Experiments were performed in three replications.

### 2.4 Mathematical model of drying curves

According to Equation (1), moisture ratios of blackberry are dependent upon the initial moisture, balanced moisture, and sample's moisture at the corresponding time (Aktaş et al., 2019).

(1) $$\text{MR}=\frac{M_{t}-M_{e}}{M_{o}-M_{e}}$$

The Guggenheim–Anderson–de Boer (GAB) model was used to calculate the equilibrium moisture content (EMC) for blackberry due to its accuracy (Giraldo Gomez et al., 2011):

(2) $$X=\frac{\left(C-1\right)Ka_{w}X_{m}}{1+\left(C-1\right)Ka_{w}}+\frac{Ka_{w}X_{m}}{1-Ka_{w}}$$

The coefficients of GAB model for blackberry at different temperatures (20–70°C) are shown in Table 1. The EMC of blackberry was obtained with the value of $M_{e}$ = 0.361 wt% db.

**TABLE 1:** Estimated coefficient values (*C*, *K*, *m*_0_) of GAB model

Temperature (°C)	*a_w_*_range	*C*	*K*	*X_m_* (kg H_2_O/kg solids)
20	0.070–0.907	11.51	0.938	0.0585
30	0.069–0.900	8.69	0.948	0.0597
40	0.066–0.893	6.91	0.955	0.0592
50	0.059–0.884	5.56	0.958	0.0580
60	0.055–0.877	4.42	0.961	0.0574
70	0.051–0.870	3.55	0.966	0.0569

The models used to fit the data gathered from drying experiment of blackberry are shown in Table 2. Moisture ratios obtained from the experiments were fitted by using MATLB R2015a software (The MathWorks, Inc.) with the listed models. Three criteria of correlation coefficient (*R* ^2^), chi‐square ( $\chi^{2}$ ), and root mean square error (RMSE) were used to determine the best fit (Torki‐Harchegani, Ghasemi‐Varnamkhasti, Ghanbarian, Sadeghi, & Tohidi, 2016).

(3) $$R^{2}=1-\frac{\sum_{i=1}^{N}{\left[{\text{MR}}_{\exp,i}-{\text{MR}}_{\text{pre},i}\right]}^{2}}{\sum_{k=1}^{N}{\left[\frac{\sum_{k=1}^{n}{\text{MR}}_{\text{pre},i}}{N}-{\text{MR}}_{\text{pre},i}\right]}^{2}}$$

(4) $$\chi^{2}=\frac{\sum_{i=1}^{N}{\left(MR_{\exp,i}-MR_{\text{pre},i}\right)}^{2}}{N-z}$$

(5) $$\text{RMSE}={\left[\frac{1}{N}\sum_{i=1}^{N}{\left({\text{MR}}_{\text{pre},i}-{\text{MR}}_{\exp,i}\right)}^{2}\right]}^{\frac{1}{2}}$$

**TABLE 2:** Applied models to fit the experimental data

References	Equations	Models
Szadzińska et al. (2019)	MR = exp(−*kt*)	Newton (Lewis)
Mghazli et al. (2017)	MR = *a* exp(−*kt*)	Henderson and Pabis
Silva et al. (2019)	MR = exp(−*kt^n^*)	Page
Şahin and Ozturk (2018)	MR = *a* exp(−*kt*) + c	Logarithmic
Behera and Sutar (2018)	MR = 1 + *at *+ *bt* ^2^	Wang and Singh
Tao et al. (2018) and Midilli, Kucuk, and Yapar (2002)	MR = *a* exp(−*kt^n^*) + *bt*	Midilli et al.
Chayjan, Kaveh, and Khayati (2017)	MR = *a*/(1 + *b* exp(*kt*)	Logistic
Kaveh et al. (2018)	MR = *a* exp(−*kt^n^*) + *bt*	Demir et al.

### 2.5 Determining *D*_eff_ and *E_a_*

The $D_{\text{eff}}$ and $E_{a}$ are considered very important to be used in modeling and designing of drying and the mass transfer processes (Amiri Chayjan, Kaveh, & Khayati, 2015). Diffusion is the main process of drying of wet materials which controls the moisture's movement and involves liquid diffusion, vapor diffusion, and hydrodynamic flow. In order to balance the mass of the moisture during drying, Fick's second law of diffusion, which is quite similar to infiltration, was used (Guo, Sun, Cheng, & Han, 2017). By using the $D_{\text{eff}}$ , drying (water removal) rate can be determined. Therefore, $D_{\text{eff}}$ of blackberry can be computed by using Equation (6) (Koukouch et al., 2017):

(6) $$\frac{\partial M}{\partial t}=D_{\text{eff}}\frac{\partial^{2}M}{\partial x^{2}}$$

The second law of Fick, relating to the unstable condition in spherical forms, can describe the moisture coefficient during the drying process (Abasi, Minaei, & Khoshtaghaza, 2017):

(7) $$\text{MR}=\frac{M_{t}-M_{e}}{M_{a}-M_{e}}=\frac{6}{\pi^{2}}\sum_{n=1}^{\infty }\frac{1}{n^{2}}\exp\left(\frac{-D_{\text{eff}}n^{2}\pi^{2}t}{r^{2}}\right)$$

Equation (7) can be summarized as below for the long period of drying (Tao, Yang, Yu, & Yang, 2018):

(8) $$\text{MR}=\left(\frac{6}{\pi^{2}}\right)\exp\left(-\frac{\pi^{2}D_{\text{eff}}t}{r_{e}^{2}}\right)$$

By plotting the data obtained from the experiments, there will be a line with k slope against the time, and the equality between this slope and coefficient of t in Equation (8) leads to the possibility to calculate *D*_eff_ by using Equation (9) (Dibagar & Amiri Chayjan, 2019).

(9) $$k=\left(\frac{D_{\text{eff}}\pi^{2}}{r^{2}}\right)$$

$E_{a}$ is the minimum required energy to start the drying process under the impacts of $D_{\text{eff}}$ and drying temperature. The relationship between temperature and *D*_eff_ is presented in Equation (10) (Xie et al., 2018):

(10) $$D_{\text{eff}}=D_{0}\exp\left(\frac{E_{a}}{\mathrm{RT}}\right)$$

In addition, in order to calculate *E_a_*, a linear relation was used:

(11) $$\ln\left(D_{\text{eff}}\right)=\ln\left(D_{0}\right)-\left(\frac{E_{a}}{R}.\frac{1}{T}\right)$$

By drawing a graph of ln(*D*_eff_) versus (1/*T*), a line with a slope $K_{1}$ could be obtained as follows (Koukouch et al., 2017):

(12) $$K_{1}=\frac{E_{a}}{R}$$

### 2.6 Specific Energy Consumption (SEC)

The required energy to extract 1 kg of blackberry moisture by means of hot air dryer with different pretreatments is expressed as SEC. It is supplied from two energy sources: (a) thermal energy mainly provided by the hot air and (b) mechanical energy of the blower. The former was obtained from Equation (13) (Kaveh, Amiri Chayjan, Taghinezhad, Rasooli Sharabiani, & Motevali, 2020):

(13) $${\text{EU}}_{\text{ter}}=\left(A\cdot v\cdot C_{a}\cdot \rho_{a}\cdot \Delta T\cdot t\right)\cdot 3600$$

The mechanical energy obtained from the blower was computed by Equation (14) (Rad, Kaveh, Sharabiani, & Taghinezhad, 2018):

(14) $${\text{EU}}_{\text{mec}}=\Delta P\cdot M_{\text{air}}\cdot t$$

The ${\text{SEC}}_{\text{con}}$ for blackberry drying in hot air dryer was calculated using Equation (15) (Mohammadi, Tab atabaekoloor, & Motevali, 2019):

(15) $${\text{SEC}}_{\text{con}}=\frac{{\text{EU}}_{\left(\text{mec}+\text{ter}\right)}}{M_{W}}$$

The thermal energy in microwave dryer was computed using Equation (16) (Motevali, Minaei, Banakar, Ghobadian, & Khoshtaghaza, 2014):

(16) $${\text{EU}}_{\text{ter}}=P\cdot t\cdot 3600$$

The SEC due to the microwave pretreatment was calculated using Equation (17) below (Wang et al., 2018):

(17) $${\text{SEC}}_{\text{mic}}=\frac{{\text{EU}}_{\left(\text{mec}+\text{ter}\right)}}{M_{W}}$$

Furthermore, ultrasonic power and SEC could be approximated using Equations (18) and (19), respectively (Kaveh et al., 2018).

(18) UP=UIcosΦ

(19) $${\text{SEC}}_{\text{ult}}=\frac{\text{UP}\cdot t}{m_{\text{vult}}}$$

Finally, the SEC of hot air dryer with microwave and ultrasonic pretreatments was obtained, respectively, from Equations (20) and (21) (Abdoli, Zare, Jafari, & Chen, 2018).

(20) $${\text{SEC}}_{\text{con}+\text{mic}}={\text{SEC}}_{\text{con}}+{\text{SEC}}_{\text{mic}}$$

(21) $${\text{SEC}}_{\text{con}+\text{ult}}={\text{SEC}}_{\text{con}}+{\text{SEC}}_{\text{ult}}$$

The SEC for ascorbic acid pretreatment was very low, and hence, it could be neglected. This pretreatment basically effects the drying time, leading to some changes in texture and further influences the drying energy consumption. Moreover, to calculate the SEC of blanching pretreatment, SEC calculation of the hot air dryer method was employed.

### 2.7 Color

To measure the color, the dried samples were firstly scanned using the scanner device (HP Scanjet G3110) having 540 × 390 pixel resolution. After scanning, three samples from each repeated experiment were selected, and then, five points, including top, bottom, left, right, and central points, from each sample were selected and analyzed (Golpour & Chayjan, 2014). Finally, from the average values including 15 points from each repetition, color, and *∆E* indexes were obtained. ΔE was gathered and recorded from digital photos after and before drying as indexes, such as *L* for brightness, *a* for redness–greenness, and *b* for yellowness–blueness. In order to observe the color changes during drying (color difference of samples from fresh blackberry), ΔE index was adopted, as shown in Equation (22) (Lagnika et al., 2019):

(22) $$\Delta E=\sqrt{{\left(L-L_{0}^{*}\right)}^{2}+{\left(a-a_{0}^{*}\right)}^{2}+{\left(b-b_{0}^{*}\right)}^{2}}$$

### 2.8 Shrinkage

Shrinkage is considered as the ratio of the final volume of the dried to the initial volume of the undried products. The extent of shrinkage is influenced by the method and degree of the product's drying. Shrinkage happens when water is removed from the cell space and the air is replaced instead (Tsuruta, Tanigawa, & Sashi, 2015). Sample's shrinkage was determined in specific mass experiments by means of toluene and represented as Equation (23) (Udomkun & Innawong, 2018).

(23) $$S_{b}=\frac{\left(V_{0}-V\right)}{V_{0}}\times 100$$

## 3 RESULTS AND DISCUSSION

### 3.1 Drying characteristic

**FIGURE 2:**
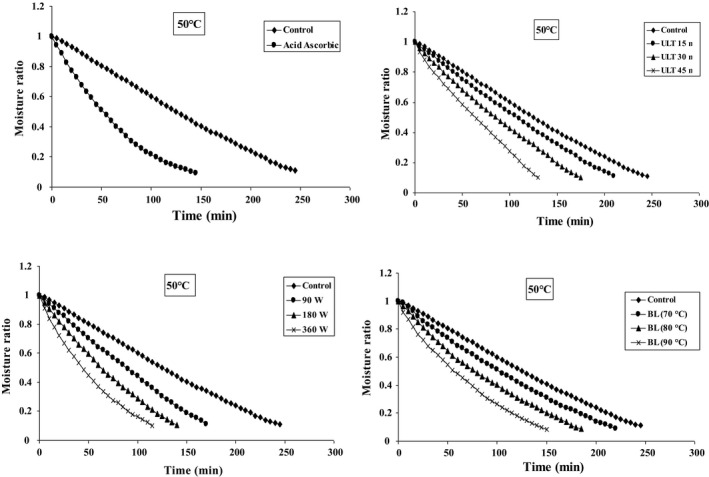
Effect of different pretreatments on variations moisture ratio versus drying time at 50°C

**FIGURE 3:**
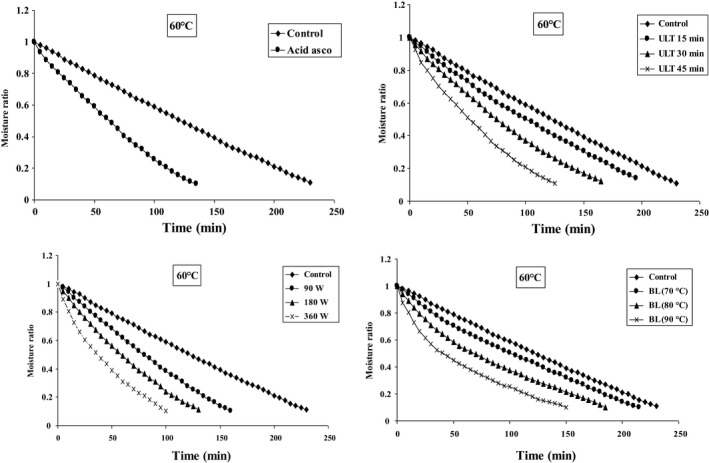
Effect of different pretreatments on variations moisture ratio versus drying time at 60°C

**FIGURE 4:**
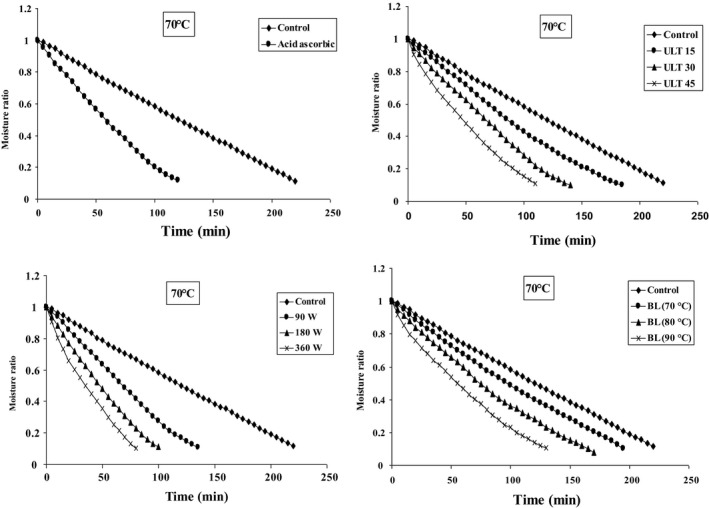
Effect of different pretreatments on variations moisture ratio versus drying time at 70°C

Figures 2, 3, 4 represent the data collected at different air temperatures (50–70°C) and various pretreatment conditions (blanching, microwave, ultrasonic, and ascorbic acid) for drying of blackberry. The moisture content was constantly decreasing following the progress of drying time. In addition, the application of pretreatment potentially shortens the drying time. The increase of temperature in different pretreatments accelerates the moisture transfer from material texture to its surface, as well as increases the evaporation rate (Ricce, Rojas, Miano, Siche, & Augusto, 2016). Generally, the pretreated samples show higher moisture transport rate (moisture discharge from the center to the surface of the material) compared to the control samples (Soghani, Azadbakht, & Darvishi, 2018). Texture density has decreased due to the adoption of pretreatments, and therefore, the moisture is exhausted from blackberry layer with faster speed (Dibagar & Amiri Chayjan, 2019).

Ultrasonic pretreatment, by means of application of cavitation, causes extreme expansion and contraction which makes the structure of a product similar to that of a sponge. Formation of this spongy texture leads to faster moisture removal and evaporation compared to the control condition (da Silva et al., 2019). Furthermore, the microwave pretreatment also reduces the drying time. The results show that the minimum drying time, which is 80 min, could be achieved under microwave pretreatment with a power of 360 W and inlet air temperature of 70°C. On the other hand, the longest drying time was 245 min using the control samples at inlet air temperature of 50°C.

Comparing these four different pretreatments, drying with microwave pretreatment potentially led to the shortest drying time. It is considered that microwave pretreatment damages the surface layer of blackberry causing pores enlargement. Therefore, the moisture inside the material is immediately transported to the surface layer and evaporated (Deepika & Sutar, 2018). Similar results have been reported in drying of parsley leaves (Sledz et al., 2016), white mushroom (Soghani et al., 2018), red apples (Doymaz, 2010), strawberry (Amami et al., 2017), and cauliflower (Sahin & Doymaz, 2017).

### 3.2 Modeling of drying kinetic

After calculating the moisture ratio under different drying temperatures and pretreatments, the models listed in Table 2 were fitted on the curves derived from the experimental data. Furthermore, different forms of drying were evaluated according to the rate of *R* ^2^, RMSE , and $\chi^{2}$ . The models were compared and selected according to higher *R* ^2^ values, and lower RMSE and $\chi^{2}$ values. Results of experimental data fitted with the presented models are shown in Table 3. The evaluation of statistical parameters shows that* R* ^2^, RMSE , sand $\chi^{2}$ ranged from 0.9929 to 0.9996, 0.01495 to 0.11236, and 0.00037 to 0.00466, respectively. It is shown that almost all the models were appropriate. However, the model developed by Midilli et al. (2002) showed the highest *R* ^2^, lowest RMSE , and $\chi^{2}$ . Therefore, this model was considered as the best model to predict the drying rate of blackberry with the high accuracy.

**TABLE 3:** Different drying models with *R* ^2^, RMSE, and *χ* ^2^ values

Models	Pretreatment	*R* ^2^	RMSE	*χ* ^2^
Newton	Control	0.9939	0.08857	0.00405
	Blanching	0.9945	0.07333	0.00331
	Microwave	0.9938	0.08902	0.00411
	Ultrasound	0.9929	0.11236	0.00466
	Acid Ascorbic	0.9940	0.08420	0.00397
Henderson and Pabis	Control	0.9948	0.06292	0.00315
	Blanching	0.9952	0.05921	0.00278
	Microwave	0.9955	0.05215	0.00267
	Ultrasound	0.9935	0.09129	0.00399
	Acid Ascorbic	0.9940	0.08129	0.00372
Page	Control	0.9967	0.03021	0.00199
	Blanching	0.9971	0.02182	0.00189
	Microwave	0.9979	0.01846	0.00148
	Ultrasound	0.9950	0.06235	0.00289
	Acid Ascorbic	0.9962	0.04018	0.00223
Logarithmic	Control	0.9966	0.03459	0.00203
	Blanching	0.9977	0.01869	0.00159
	Microwave	0.9971	0.02167	0.00187
	Ultrasound	0.9959	0.04236	0.00249
	Acid Ascorbic	0.9970	0.02222	0.00193
Wang and Singh	Control	0.9978	0.01845	0.00151
	Blanching	0.9965	0.03499	0.00207
	Microwave	0.9981	0.01781	0.00131
	Ultrasound	0.9972	0.02125	0.00180
	Acid Ascorbic	0.9976	0.01901	0.00163
Midilli et al.	Control	0.9992	0.01585	0.00067
	Blanching	0.9990	0.01622	0.00079
	Microwave	0.9996	0.01495	0.00037
	Ultrasound	0.9988	0.01652	0.00090
	Acid Ascorbic	0.9995	0.01515	0.00043
Logistic	Control	0.9984	0.01735	0.00114
	Blanching	0.9990	0.01615	0.00078
	Microwave	0.9992	0.01597	0.00070
	Ultrasound	0.9980	0.01790	0.00135
	Acid Ascorbic	0.9988	0.01669	0.00092
Demir et al.	Control	0.9982	0.01765	0.00127
	Blanching	0.9980	0.01799	0.00138
	Microwave	0.9985	0.01712	0.00108
	Ultrasound	0.9979	0.01822	0.00144
	Acid Ascorbic	0.9983	0.01752	0.00121

### 3.3 Effects on *D*_eff_ and *E_a_*

#### 3.3.1 Effect of blanching pretreatment

As shown in Table 4, the highest $D_{\text{eff}}$ , which is 6.30 × 10^–9^ m^2^/s, was achieved at inlet air temperature of 70°C and blanching pretreatment of 90°C. On the other hand, the minimum $D_{\text{eff}}$ (3.20 × 10^–9^ m^2^/s) was obtained at inlet air temperature of 50°C and blanching pretreatment of 70°C. The results show that by increasing the blanching pretreatment temperature, $D_{\text{eff}}$ increases. It is considered that by applying the blanching pretreatment, the membrane resistance of cells is destructed due to high temperature, and therefore, the moisture can moves easily from the inside to the outside of the material, leading to the increase of $D_{\text{eff}}$ (Akonor & Tortoe, 2014). Raising the temperature of blanching pretreatment from 70 to 90°C leads to an increase of membrane layer destruction, and the product texture gets more destructed following the fast removal of moisture.

**TABLE 4:** Effective moisture diffusion coefficient and activation energy using different pretreatments

Pretreatment	Temperature (°C)	*D*_eff_ (m^2^/s)	*E*_a_ (kJ/mol)
Control	50	3.10 × 10^–9^	13.61
	60	3.27 × 10^–9^	
	70	3.42 × 10^–9^	
Ascorbic acid	50	6.32 × 10^–9^	17.65
	60	6.45 × 10^–9^	
	70	6.65 × 10^–9^	
Ultrasonic 15 min	50	3.30 × 10^–9^	21.06
	60	3.60 × 10^–9^	
	70	4.28 × 10^–9^	
Ultrasonic 30 min	50	4.24 × 10^–9^	23.17
	60	4.69 × 10^–9^	
	70	5.66 × 10^–9^	
Ultrasonic 45 min	50	6.43 × 10^–9^	24.24
	60	6.70 × 10^–9^	
	70	6.98 × 10^–9^	
Microwave 90 W	50	4.60 × 10^–9^	24.79
	60	5.17 × 10^–9^	
	70	6.11 × 10^–9^	
Microwave 180 W	50	6.68 × 10^–9^	25.02
	60	7.13 × 10^–9^	
	70	8.07 × 10^–9^	
Microwave 360 W	50	7.20 × 10^–9^	23.64
	60	8.05 × 10^–9^	
	70	1.00 × 10^–8^	
Blanching 70°C	50	3.20 × 10^–9^	22.53
	60	3.36 × 10^–9^	
	70	3.69 × 10^–9^	
Blanching 80°C	50	4.06 × 10^–9^	24.15
	60	4.49 × 10^–9^	
	70	5.08 × 10^–9^	
Blanching 90°C	50	5.88 × 10^–9^	26.08
	60	6.10 × 10^–9^	
	70	6.30 × 10^–9^	

Blanching pretreatment at different temperatures also leads to the change of $E_{a}$ , ranging from 22.53 to 26.26 kJ/mol. Agarry (Agarry, 2016) found that $D_{\text{eff}}$ of tomato slices with ultrasonic and blanching pretreatments were 4.43 × 10^–11^ and 6.33 × 10^–9^ m^2^/s, respectively. Moreover, Akonor and Tortoe (Akonor & Tortoe, 2014) have calculated $D_{\text{eff}}$ of chayote drying with hot air and blanching pretreatment, which was 1.30 × 10^–8^ m^2^/s. Doymaz (2010) have studied the drying of red apple using hot air drying, with the addition of citric acid and blanching pretreatment. The results showed that $D_{\text{eff}}$ ranged between 2.93 × 10^–10^ and 6.06 × 10^–10^, and $E_{a}$ obtained for the control sample, blanching pretreatment, and citric acid pretreatment were 22.06, 18.93, and 14.47 kJ/mol, respectively. Other researchers studied cauliflower drying with blanching pretreatment and hot air, and found that $D_{\text{eff}}$ ranged from 7.90 × 10^–9^ to 1.88 × 10^–8^ m^2^/s and $E_{a}$ was 29.09 kJ/mol. (Sahin & Doymaz, 2017).

#### 3.3.2 Effect of microwave pretreatment

As shown in Table 4, in case of microwave pretreatment, the maximum $D_{\text{eff}}$ was 1.00 × 10^–8^ which was achieved under microwave power and air temperature of 360 W and 70°C, respectively. Furthermore, the minimum $D_{\text{eff}}$ was 4.60 × 10^–9^ m^2^/s, earned under conditions of microwave power of 90 W and air temperature of 50°C. It can be observed that the increase of microwave power results in the increase of $D_{\text{eff}}$ . One of the main reasons for this phenomenon is that the microwave influences the blackberry texture leading to an increase in porosity and opening of the capillary tubes. Therefore, the moisture transport becomes faster and the drying time can be reduced.

On the other hand, as microwave power increases, the heating rate of the sample increases accordingly due to the increase of electric dipole intensity of water. As the result, heat generation inside the sample increases leading to an intensive pressure difference between center and surface of the molecule (Behera & Sutar, 2018). The product texture in pretreatment with higher power suffers larger disruption and divergence, and $D_{\text{eff}}$ increases (Horuz, Bozkurt, Karataş, & Maskan, 2018). $E_{a}$ by means of microwave pretreatment ranged from 23.64 to 24.79 kJ/mol. Mierzwa, Szadzińska, Pawłowski, Pashminehazar, and Kharaghani (2019) have studied raspberry drying using hot air and adopted microwave and ultrasonic pretreatments, and obtained $D_{\text{eff}}$ ranging from 0.17 × 10^–8^ to 3.91 × 10^–8^ m^2^/s. They also showed that the effect of microwave pretreatment on $D_{\text{eff}}$ is more significant compared to ultrasonic pretreatment. In addition, the impacts of various pretreatments (blanching, microwave, and pulse electric field) on okra drying also have been investigated by Adedeji et al. (Adedeji et al., 2008). They showed that the highest $D_{\text{eff}}$ has been achieved by microwave pretreatment, while the lowest one was obtained by the control sample (Adedeji et al., 2008).

#### 3.3.3 Effect of ultrasonic pretreatment

As presented in Table 4, the highest and lowest $D_{\text{eff}}$ for ultrasonic treatment were 3.30 × 10^–9^ and 6.98 × 10^–9^ m^2^/s, respectively. Furthermore, drying temperature and duration of ultrasonic pretreatment also had significant impact on $D_{\text{eff}}$ values. The maximum $D_{\text{eff}}$ was obtained in conditions of air temperature of 70°C and pretreatment duration of 45 min. By increasing the temperature, the movement of water molecules is intensified leading to faster moisture transfer and removal, therefore, the drying time decreases while $D_{\text{eff}}$ increases (Bozkir, Ergün, Tekgül, & Baysal, 2019). On the other hand, ultrasonic pretreatment also leads to the opening of capillary tube because of scattering surface compounds and formation of long microscopic channels (Motevali & Zabihnia, 2017). The change in cell formation and damage of the cell walls lead to faster moisture removal. $E_{a}$ in drying with ultrasonic pretreatment ranged from 21.06 to 24.25 kJ/mol. Kaveh et al. (2018) have performed the drying of almonds and found that $D_{\text{eff}}$ in case of drying with ultrasonic pretreatment was between 1.81 × 10^–9^ and 9.70 × 10^–9^ m^2^/s. In addition, they also calculated $E_{a}$ which ranged from 26.35 to 36.44 kJ/mol. Moreover, in the case of strawberry drying with ultrasonic pretreatment, Amami et al. (2017) found the value of $D_{\text{eff}}$ was between 8.42 × 10^–10^ and 25.96 × 10^–10^ m^2^/s depending on the pretreatment duration. They showed that $D_{\text{eff}}$ increased following the increase of air temperature, viscosity of osmotic solution, and duration of ultrasonic application. This is due to the increase of thermal gradient following the rise of temperature and surface evaporation.

#### 3.3.4 Effect of ascorbic acid pretreatment

Table 4 also shows the lowest $D_{\text{eff}}$ in the control sample, which is 3.10 × 10^–9^ m^2^/s. By adopting ascorbic acid pretreatment, these values changed to 6.65 × 10^–9^ m^2^/s for the highest and 6.32 × 10^–9^ m^2^/s for the lowest values. Increasing the drying temperature leads to the increase of $D_{\text{eff}}$ compared to the control sample. The temperature, together with ascorbic acid pretreatment, strongly influences the molecule movement and surface suction, leading to the increase of $D_{\text{eff}}$ (Sahin & Doymaz, 2017). On the other hand, $E_{a}$ in control sample was 13.61 kJ/mol and increased to 17.65 kJ/mol when the ascorbic acid pretreatment was adopted. It is clear that ascorbic acid pretreatment leads to the increase of $E_{a}$ . This is due to creation of microscopic ducts and change in product texture with preservation of its structure. Therefore, the energy for drying can be reduced (Motevali & Zabihnia, 2017). Furthermore, compared to the control sample, the application of different types of pretreatments on red apple led to the increase of $D_{\text{eff}}$ . $D_{\text{eff}}$ value for blackberry in solar drying was 9.66 × 10^–9^ (López‐Ortiz et al., 2018). The lowest and highest $D_{\text{eff}}$ values for blackberry in infrared drying were estimated to be 1.14 × 10^–9^ and 3.08 × 10^–9^ m^2^/s, respectively (Kıpçak, 2019).

### 3.4 SEC

Energy consumption is known to be one of the main parameters of evaluation during process operations, including drying and distillation. Because of the lack of fossil fuels and environmental problems (greenhouse gas emission), energy price is still increasing and fluctuating, leading to urgent need for new strategies to decrease energy consumption in industry (Mierzwa et al., 2019).

**FIGURE 5:**
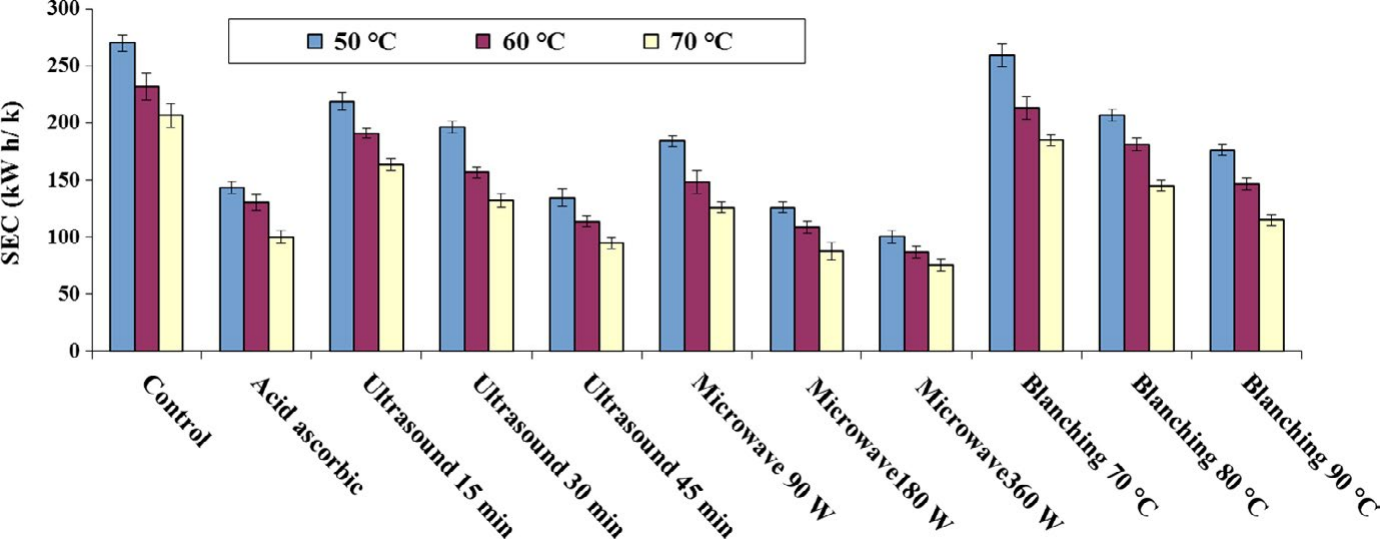
Specific energy consumption of blackberry in hot air drying with different pretreatments

Figure 5 represents the SEC of blackberry drying for the control sample and different pretreatments. It is clear that by introducing ultrasonic (duration of 15–45 min), blanching (duration of 70–90°C), microwave (power of 90–360 W), and also ascorbic acid pretreatments, the SEC decreases compared to the control sample.

As presented in Figure 5, the lowest SEC for blackberry drying was obtained under microwave pretreatment, which is 75.63 kW hr/kg, with the conditions of microwave power of 360 W, and inlet air temperature of 70°C. On the other hand, the highest SEC (269.91 kW hr/kg) was obtained in the drying of the control sample with the drying temperature of 50°C. Microwave treatment leads to temperature increase within the sample, resulting in water molecules polarization and porous formation with larger diameter. This also avoids any superficial hardening during drying and leads to better moisture removal from the sample, and hence, the energy consumption can be reduced (Torki‐Harchegani et al., 2016). In addition, by increasing the inlet air temperature of all pretreatments, the SEC decreases. As expected, by increasing the temperature, larger, and faster moisture removal from sample can be realized, hence, drying time and SEC decrease accordingly. In the other words, higher inlet temperature leads to larger mass transfer (Filippin, Molina Filho, Fadel, & Mauro, 2018). In drying of raspberry with different pretreatments, the lowest SEC was obtained under microwave pretreatment (Mierzwa et al., 2019). Furthermore, Szadzińska, Łechtańska, Pashminehazar, Kharaghani, and Tsotsas (2019) suggested that using different pretreatments in the raspberries drying under hot air dryer improved the drying rate and thus reduced the SEC. They also showed that the highest and lowest SECs for the control sample and microwave pretreatment were 0.8 and 0.37 MJ/g, respectively.

### 3.5 Color

**FIGURE 6:**
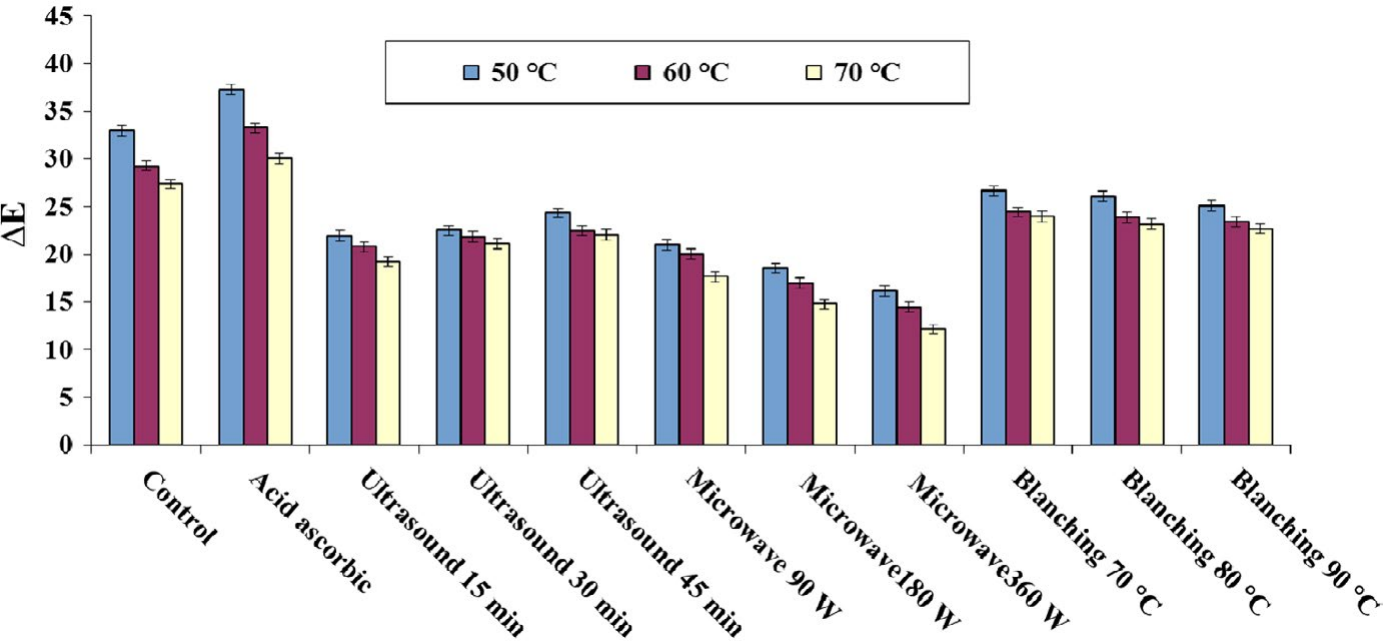
Color changes of blackberry in hot air drying under different pretreatments

The color preservation of biological products is as an important quality index to evaluate the damage caused by the thermal process. Color is a major concern for product acceptance by consumers (Aral & Beşe, 2016). Figure 6 shows the correlation of temperature, pretreatment method, and color index ( ΔE ). The highest color change (37.27) happened in ascorbic acid pretreatment because of pigment decomposition, enzymatic reactions, and nonenzymatic browning reactions (Wang et al., 2018). On the other hand, the minimum color change was found in microwave pretreatment (12.15) with a power of 360 W and inlet air temperature of 70°C. Lower change in the color index leads to better color preservation. Therefore, it can be stated that the best quality of blackberry color can be achieved under microwave pretreatment with higher drying temperature, which can be attributed as fast drying operation (Zielinska, Zapotoczny, Alves‐Filho, Eikevik, & Blaszczak, 2013).

The increase in pretreatment duration using ultrasonic leads to the increase of color change. It is considered that the color change during blackberry drying happens because of various factors, including thermal destruction of carotenoid, oxidation, enzymatic reactions, and nonenzymatic browning reactions (Chen, Guo, & Wu, 2016). These results are similar with other researches, including Çağlayan & Barutçu Mazı (2018) for pumpkin drying, Ren, Perussello, Zhang, Kerry, and Tiwari (2018) for onion drying, Amami et al. (2017) for strawberry drying, and Lagnika et al. (2019) for sweet potato drying (Amami et al., 2017; Çağlayan & Barutçu Mazı, 2018; Lagnika et al., 2019; Ren et al., 2018).

Furthermore, by increasing the temperature in blanching pretreatment, the color change decreases accordingly. The largest color change happened at 70°C temperature and 3 min immersion, in which the color removal is assumed to be caused by high steam temperature. These changes can be made by creation of free radicals and sono‐chemical due to cavitation, which may affect the food properties (Ren et al., 2018). In spray drying of blackberry, Weber et al. (Weber, Boch, & Schieber, 2017) found that the highest and lowest color changes were 24 and 7, respectively. In addition, in blackberry drying using a spray drying with different temperatures (110–170°C), it was observed that color changes decreased following the increase of air temperature (Cervantes et al., 2016).

### 3.6 Shrinkage

**FIGURE 7:**
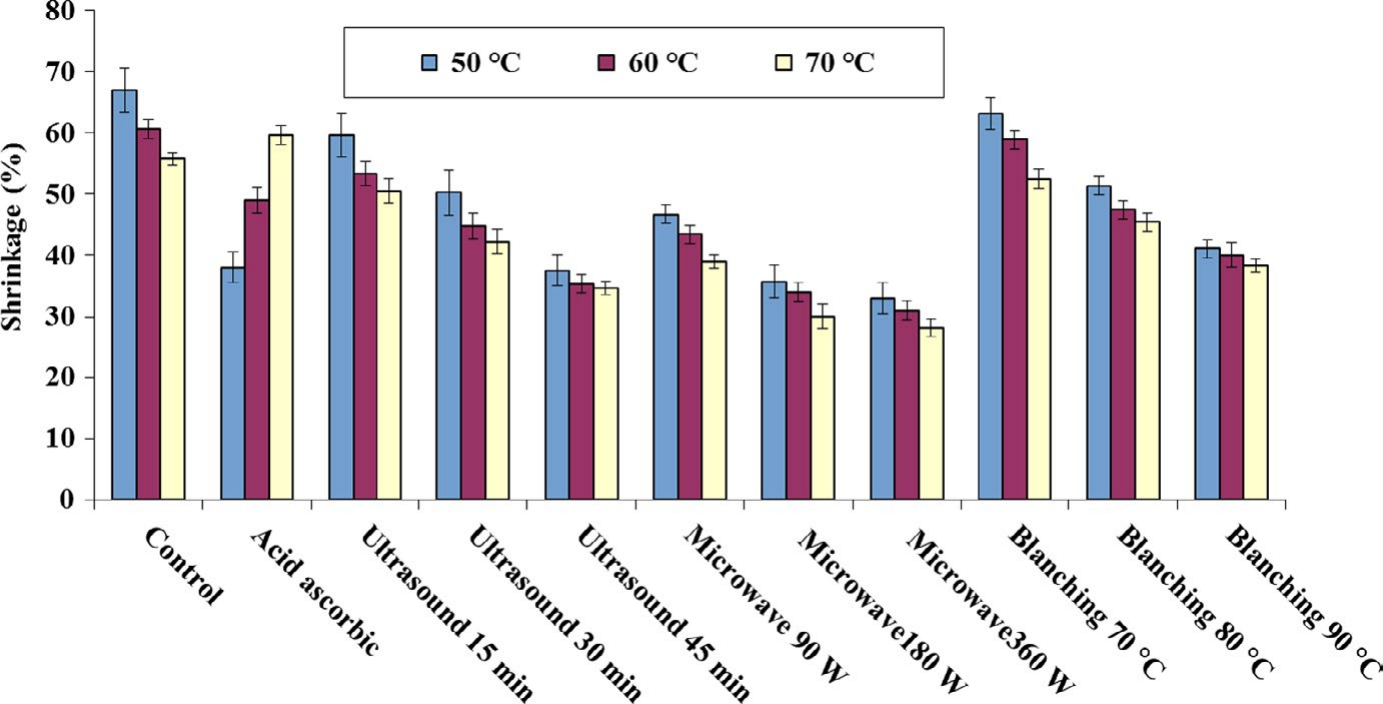
Shrinkage of blackberry in hot air drying under different pretreatments

Shrinkage affects the physical properties, such as porosity, density, and product shape. As water removal increases, shrinkage increases accordingly (Parthasarathi & Anandharamakrishnan, 2014). Drying of blackberry by means of different pretreatments leads to the shrinkage from 28.12% to 66.97%, as shown in Figure 7. The shrinkage caused by ascorbic acid, microwave, ultrasonic, and blanching pretreatments were 38.03%–59.62%, 28.12%–46.67%, 34.64%–59.62%, and 38.34%–63.12%, respectively. For reference, drying of the control sample without pretreatment resulted in the shrinkage of 55.77%–66.97%. The largest shrinkage happened in the control sample at a temperature of 50°C, which was 66.97%. On the other hand, the smallest shrinkage was obtained in the microwave pretreatment with a power of 360 W and air temperature of 70°C, which was 28.12%.

The water steam pressure was created during drying with microwave pretreatment, causing cells expansion, which is known as puffy effect. This phenomenon together with the microwave heating can reduce the shrinkage (Aydogdu, Sumnu, & Sahin, 2015). According to Figure 7, with the increase in microwave power, duration of ultrasonic application, duration of blanching, and inlet air temperature, the amount of shrinkage was reduced. This is due to faster moisture transfer to the outer layer of samples after the application of different pretreatments before drying (Dehghannya, Bozorghi, & Heshmati, 2018). These results are coherent with the results of chokeberry drying (Calín‐Sánchez et al., 2015), potato cubes drying (Dehghannya et al., 2018), raspberry drying (Mierzwa et al., 2019), and chayote drying (Akonor & Tortoe, 2014).

## 4 CONCLUSION

Drying is one of the most commonly used methods to reduce the product moisture, especially for long period of storage and transportation. In this research, the effects of blanching, microwave, ultrasonic, and ascorbic acid pretreatments on drying performances (drying time, $D_{\text{eff}}$ , $E_{a}$ , and SEC) and quality (color and shrinkage) of blackberry were studied. Eight mathematical models were adopted to predict the moisture ratio of blackberry. The results proved that the application of these pretreatments on the samples generally shortens the drying time.

Microwave pretreatment shows the most significant effect on drying. In addition, the fitting of mathematical models to the drying data showed that the model developed by Midilli et al. (2002) matches best for the control sample and the four different pretreatments. Moreover, $D_{\text{eff}}$ shows increasing effect with ascorbic acid pretreatment. However, $D_{\text{eff}}$ increases following ultrasonic application duration, blanching temperature, and microwave power. The highest SEC was obtained in case the control sample, and the application of different pretreatments causes the reduction of SEC. Finally, the lowest color change and shrinkage were achieved in microwave pretreatment with power of 360 W and drying air temperature of 70°C.

## Nomenclature

*A*: Cross‐sectional area of the sample tray (m^2^)
$a_{w}$: Water activity of blackberry
C: Constant related to the heat of sorption of the first layer
$C_{a}$: Input or output air specific heat (kJ/kg°C)
cosΦ: Power factor considered to be 0.8
$D_{\text{eff}}$: Effective moisture diffusivity (m^2^/s)
*D*_0_: Intercept which is constant
*E_a_*: Activation energy (kJ/mol)
${\text{EU}}_{\text{mec}}$: Mechanical energy consumption (kJ)
${\text{EU}}_{\text{ter}}$: Thermal energy consumption (kJ)
*K*: Constant related to the heat of sorption of the multilayer
$M_{t}$: Moisture content (wt% db)
$M_{o}$: Initial moisture content (wt% db)
$M_{e}$: Equilibrium moisture content (wt% db)
MR: Moisture ratio (‐)
$M_{W}$: Mass transfer amount in (kg) for microwave
${\text{MR}}_{\exp,i}$: Experimental values
${\text{MR}}_{\text{pre},i}$: Predicted values by calculating from the model for this measurements
$m_{\text{vult}}$: Mass transfer amount (kg) for ultrasonic
*N*: Number of observations
*n*: Number of terms taken into consideration
*I*: Current applied to the ultrasonic generator (A)
*P*: Microwave power (kW)
*R*: Radius of the samples (m)
*R*: Universal gas constant (equal to 8.3143 kJ/mol)
${\text{SEC}}_{\text{mic}}$: Specific energy consumption for microwave (kWh/kg)
${\text{SEC}}_{\text{ult}}$: Specific energy consumption for ultrasonic (kWh/kg)
$S_{a}$: Shrinkage percentage (%)
*T*: Temperature inside the drying chamber (K)
t: Drying time (s)
$V_{0}$: Initial volume (m^3^)
V: Secondary volume or volume after drying (m^3^)
*v*: Input air velocity (m/s)
UP: Ultrasonic power (kW)
U: Voltage applied to the ultrasonic generator (V)
X: Equilibrium moisture content (kg‐H_2_O/kg‐solids)
$X_{m}$: Nanolayer moisture content (kg‐H_2_O/kg‐solids)
z: Number of constants
*ρ_a_*: Air density (kg/m^3^)
$\Delta L^{*}$: Brightness before and after drying the pistachio
$\Delta a^{*}$: Brightness of the red color
$\Delta b^{*}$: Brightness of the yellow color
ΔE: Total color change
ΔP: Different pressure (mbar)

## CONFLICT OF INTEREST

The authors declare that they do not have any conflict of interest.

## ETHICAL APPROVAL

This study does not involve any human or animal testing.
